# 

**Published:** 1890-08

**Authors:** 


### MISSISSIPPI VALLEY MEDICAL ASSOCIATION.

The seventeenth annual session of the Mississippi Valley Medical Association will be held at Louisville, Ky., October 8th, 9th and 10th, 1890. The meeting promises to be of great social and scientific interest, as the profession of Louisville are doing their utmost to make it a success. Ladies accompanying physicians will be made especially welcome. Gentlemen desiring to read papers will please send titles of the same to the Secretary, Dr. E. S. McKee, Cincinnati, at an early date.

